# Management of Vasomotor Symptoms in Women With Breast Cancer: From Pathophysiology to the Emerging Role of Neurokinin Receptor Antagonism

**DOI:** 10.7759/cureus.117665

**Published:** 2026-10-09

**Authors:** Kanelina Bimpa, Vasileios Emmanouil, Emmanouil M Xydias, Dimitrios Varvaras, Elias Tsakos

**Affiliations:** 1 Department of Breast Health, BreastCare Outpatient Clinic, Thessaloniki, GRC; 2 Department of Breast Health, EmbryoClinic IVF, Thessaloniki, GRC; 3 Department of Obstetrics and Gynecology, Komotini General Hospital "Sismanogleio", Komotini, GRC; 4 Department of Obstetrics and Gynecology, EmbryoClinic IVF, Thessaloniki, GRC; 5 Department of Breast Surgery, Breast Health Clinic Pio XI, Rome, ITA

**Keywords:** breast cancer, fezolinetant, hot flashes, kndy neurons, menopause, neurokinin-3 receptor antagonist, vasomotor symptoms

## Abstract

Vasomotor symptoms (VMS), encompassing hot flashes and night sweats, are among the most frequent and disruptive consequences of estrogen withdrawal in women with breast cancer. In this population, menopause may occur naturally or in association with cancer treatment, while the use of menopausal hormone therapy (MHT) is limited by concerns regarding oncological safety. The present narrative review synthesizes current understanding of the neuroendocrine pathophysiology of vasomotor symptoms, centered on the hypertrophy and hyperactivity of hypothalamic neurons co-expressing kisspeptin, neurokinin B (NKB), and dynorphin (KNDy) following estrogen deprivation, and critically appraises the available non-hormonal management options, including selective serotonin reuptake inhibitors, serotonin-norepinephrine reuptake inhibitors, gabapentin, oxybutynin, and cognitive behavioral therapy (CBT). Particular emphasis is placed on the neurokinin receptor antagonists, a mechanistically targeted drug class that acts on the thermoregulatory pathways involved in VMS. Fezolinetant is a selective neurokinin-3 receptor antagonist evaluated in the SKYLIGHT program, while elinzanetant is a dual neurokinin-1 and neurokinin-3 receptor antagonist evaluated in the OASIS program, including a pivotal placebo-controlled trial in women experiencing vasomotor symptoms associated with endocrine therapy for breast cancer. Dedicated studies of fezolinetant in women with breast cancer are ongoing. Neurokinin receptor antagonism represents a promising non-hormonal therapeutic strategy for VMS in this population, although agent-specific safety considerations and the need for longer-term oncological safety data require further evaluation.

## Introduction and background

Breast cancer is the most frequently diagnosed malignancy in women worldwide, with more than 2.2 million new cases reported in 2022 [1]. Adjuvant endocrine therapy plays a central role in the management of hormone receptor-positive breast cancer and may include tamoxifen, aromatase inhibitors, and ovarian function suppression, depending on menopausal status and recurrence risk [2,3]. Despite its established oncological benefit, endocrine therapy is frequently accompanied by adverse effects related to estrogen deprivation or estrogen receptor modulation. Vasomotor symptoms (VMS), including hot flashes and night sweats, are among the most common and troublesome of these effects and may be particularly pronounced in premenopausal women undergoing ovarian function suppression or in women who experience treatment-induced menopause [2,3]. These symptoms can adversely affect sleep and quality of life and may contribute to difficulties with adherence and persistence with endocrine therapy [3,4]. This is clinically relevant because non-adherence and premature discontinuation of adjuvant endocrine therapy have been associated with poorer breast cancer outcomes [4].

Effective management of VMS is therefore an important component of survivorship care. Menopausal hormone therapy (MHT) remains the most effective treatment for VMS in the general menopausal population, but systemic hormone therapy is generally contraindicated in women with hormone receptor-positive breast cancer because of concerns regarding oncological safety [5,6]. Consequently, management in this population relies predominantly on non-hormonal approaches. Established options include selective serotonin reuptake inhibitors and serotonin-norepinephrine reuptake inhibitors, gabapentin, oxybutynin, and behavioral interventions, although differences in efficacy, tolerability, drug interactions, and adverse effect profiles may limit their use in individual patients [7].

Growing understanding of the hypothalamic kisspeptin, neurokinin B (NKB), and dynorphin (KNDy) neuronal network has led to the development of neurokinin receptor antagonists as a mechanistically targeted, non-hormonal approach to VMS [8]. Fezolinetant, a selective neurokinin-3 receptor antagonist, and elinzanetant, a dual neurokinin-1 and neurokinin-3 receptor antagonist, have demonstrated efficacy in phase 3 trials for menopausal VMS [9,10]. More recently, elinzanetant has also demonstrated efficacy in women with VMS associated with endocrine therapy for hormone receptor-positive breast cancer or its prevention [11]. The present narrative review examines the epidemiology and neuroendocrine pathophysiology of VMS, reviews established non-hormonal management strategies in women with breast cancer, and evaluates the emerging role of neurokinin receptor antagonism in this population.

## Review

Methods

Search Strategy and Study Selection

A literature search was conducted using PubMed/MEDLINE and ClinicalTrials.gov and was updated through May 2026. Search terms included combinations of “vasomotor symptoms,” “hot flashes,” “breast cancer,” “endocrine therapy,” “tamoxifen,” “aromatase inhibitors,” “KNDy neurons,” “neurokinin B,” “neurokinin-3 receptor,” “fezolinetant,” and “elinzanetant.” Reference lists of relevant publications were also screened to identify additional studies. Studies were selected based on their relevance to the scope of this narrative review, with particular emphasis on evidence concerning non-hormonal treatment options and neurokinin receptor antagonism. Because this was a narrative rather than a systematic review, study selection was not conducted according to a formal systematic review protocol, and no formal risk-of-bias assessment or quantitative evidence synthesis was performed.

Epidemiology of vasomotor symptoms

VMS, commonly termed the “hot flash syndrome,” constitute the cardinal manifestation of the menopausal transition and affect up to 80% of women [12-15], with variation according to cultural and ethnic background [16,17]. Approximately 40% of women report symptom onset in early perimenopause, while 60%-80% experience symptoms during late perimenopause and early postmenopause [18-22]. Longitudinal data from the Study of Women’s Health Across the Nation have demonstrated that VMS are often persistent, with a median total duration of frequent symptoms exceeding seven years and persistence for more than four years beyond the final menstrual period [23]. Although estrogen withdrawal is the principal physiological driver, flushing may also occur in several medical conditions and in association with certain medications, which should be considered in the differential diagnosis [24].

Several factors influence the likelihood and severity of VMS. Higher body mass index and active cigarette smoking are associated with greater symptom burden [25,26], while socioeconomic disadvantage has also been linked to a higher prevalence of symptoms [25]. Serum follicle-stimulating hormone (FSH) concentrations correlate positively with VMS prevalence and frequency, more strongly than estradiol concentrations themselves [18]. Differences have also been observed across racial and ethnic groups, although these patterns likely reflect a combination of biological, environmental, socioeconomic, and cultural influences on symptom experience and reporting [15,17,25]. In women with breast cancer, this background burden may be compounded by abrupt or sustained treatment-related estrogen deprivation, making VMS particularly relevant during survivorship and adjuvant endocrine therapy [11].

Pathophysiology

The principal mechanism underlying VMS is a reduction in circulating estrogen that destabilizes hypothalamic thermoregulation [27]. Symptomatic women exhibit a markedly narrowed thermoneutral zone (the core temperature range within which neither sweating nor shivering is triggered), such that small elevations in core temperature, which would pass unnoticed in asymptomatic women whose thermoneutral zone spans approximately 0.4°C, provoke an exaggerated heat dissipation response of cutaneous vasodilatation and profuse sweating [28,29]. Estrogen deficiency alone, however, is insufficient to explain the phenomenon: prepubertal girls and women with functional hypothalamic amenorrhea also maintain low estrogen concentrations yet do not experience hot flashes, indicating that it is the withdrawal of estrogen from a previously estrogen-replete system, together with additional neuroendocrine mechanisms, that generates the symptoms [30].

Early physiological studies observed that hot flash episodes occur in close temporal association with pulses of luteinizing hormone (LH) secretion, although LH itself is not causal, since flashes persist after hypophysectomy; the co-activation more likely reflects the anatomical and functional proximity of the medial preoptic thermoregulatory area to gonadotropin-releasing hormone (GnRH) neuronal populations [31,32]. A unifying neuroanatomical framework has since emerged with the characterization of hypothalamic KNDy neurons of the arcuate nucleus, which co-express kisspeptin, neurokinin B (NKB), and dynorphin, and project to the median preoptic nucleus, the principal thermoregulatory relay [8]. Estrogen normally restrains these neurons; following estrogen withdrawal, KNDy neurons undergo marked hypertrophy with increased NKB signaling through the NK3 receptor, which destabilizes thermoregulatory set points and drives inappropriate heat dissipation effector responses [8]. Direct evidence for this pathway in humans is compelling: intravenous infusion of NKB reproduces typical hot flashes in young women with normal estrogen concentrations [33], and genetic variation at the tachykinin receptor 3 (TACR3) locus, which encodes the NK3 receptor, is associated with the presence of VMS in postmenopausal women [34]. This pathway constitutes the molecular target of the neurokinin receptor antagonists discussed below, and its modulation by serotonergic and noradrenergic afferents provides a mechanistic rationale for the partial efficacy of centrally acting antidepressants [27,29].

Although the upstream endocrine perturbation differs across clinical settings, VMS appear to converge on a common estrogen-sensitive hypothalamic thermoregulatory pathway. Natural menopause, bilateral oophorectomy, and chemotherapy-induced ovarian failure involve loss or withdrawal of ovarian estrogen production, whereas aromatase inhibitors suppress estrogen synthesis, and tamoxifen alters estrogen receptor signaling. These distinct mechanisms can therefore produce a similar thermoregulatory phenotype through disruption of estrogen-sensitive hypothalamic pathways. Importantly, KNDy neurons also constitute a key component of the GnRH/LH pulse generator [8]. Consequently, the hormonal effects of neurokinin receptor antagonism depend on reproductive endocrine status: NK3 receptor blockade has not been shown to increase circulating estradiol in postmenopausal women, whereas suppression of gonadotropin secretion and ovarian hormone concentrations has been demonstrated in women with an active reproductive axis [35,36].

Clinical manifestations and severity

Hot flashes typically begin as a sudden sensation of intense heat originating in the chest and face that spreads rapidly throughout the body, accompanied by cutaneous vasodilatation, a measurable rise in skin temperature and conductance, intense diaphoresis, and palpitations, with chills and occasionally anxiety following as core temperature falls [24,29]. Individual episodes last two to four minutes, and their frequency ranges from less than one daily to hourly episodes throughout the 24-hour cycle [24]. Nocturnal manifestations, termed night sweats, cluster within the first hours of sleep and are particularly disruptive to sleep architecture, contributing to the fatigue, irritability, and impaired concentration that many symptomatic women report [28].

Beyond their immediate discomfort, VMS carry prognostic significance for long-term health, as symptom severity has been associated with impaired quality of life, subclinical cardiovascular disease, and accelerated bone turnover with loss of bone mineral density during the menopausal transition [37,38]. In clinical research and practice, severity is commonly stratified according to the Women’s Health Initiative classification, which distinguishes mild symptoms, defined as those with no impact on sleep, daily activities, or occupational functioning, from moderate symptoms, which cause mild impairment in these domains, and severe symptoms, in which frequent episodes significantly disrupt sleep, daily activity, and work [39]. It is noteworthy that although a majority of affected women describe their symptoms as severe, only an estimated 20%-30% ever seek medical attention, underscoring the extent to which this condition remains under-treated [18,24]. In clinical trials, VMS frequency and severity are commonly assessed prospectively using daily electronic hot-flash diaries, in which individual episodes are recorded and graded as mild, moderate, or severe [9].

Therapeutic approach

Management of VMS is broadly classified into hormonal and non-hormonal strategies, and treatment selection should be individualized according to several considerations that current guidance emphasizes: the patient’s subjective perception of symptom severity and the degree of interference with daily functioning; the clinical history, including age, ethnicity, and comorbidities such as cardiovascular risk factors or a personal history of breast cancer; the patient’s preference regarding hormonal treatment; and the coexistence of other menopausal complaints, such as genitourinary symptoms or mood disturbance, which may influence the choice of agent [5,7].

Menopausal Hormone Therapy and Its Contraindication in Breast Cancer Survivors

Menopausal hormone therapy (MHT) remains the most effective intervention for VMS, reducing both frequency and severity substantially, and current guidance supports its use in appropriately selected symptomatic women without contraindications [5]. In women with a history of hormone receptor-positive breast cancer, however, systemic MHT is contraindicated, a position grounded principally in the randomized HABITS trial, which compared hormone therapy with best non-hormonal management in breast cancer survivors and was terminated prematurely after an unacceptable excess of new breast cancer events emerged in the hormone therapy arm [5,6].

The therapeutic dilemma is sharpened by the fact that breast cancer survivors are precisely the group most susceptible to severe VMS. Estrogen withdrawal in this population is frequently abrupt, resulting from cytotoxic chemotherapy-induced ovarian failure or bilateral oophorectomy rather than from the gradual decline of natural menopause, and adjuvant endocrine therapy then sustains or aggravates the symptoms for 5-10 years [11]. Tamoxifen, a selective estrogen receptor modulator with anti-estrogenic activity in the breast, and aromatase inhibitors, including the irreversible steroidal agent exemestane, are both well documented to precipitate or intensify VMS, which in turn constitute a leading contributor to premature discontinuation of endocrine therapy and thereby threaten oncological outcomes [11,40]. Susceptibility to tamoxifen-induced hot flashes additionally varies among individuals, and pharmacogenetic variation, including polymorphisms affecting estrogen receptor signaling and cytochrome P450 2D6 (CYP2D6)-mediated drug metabolism, appears to modulate both symptom expression and treatment response [41].

Non-hormonal Pharmacotherapy: Established Options

The 2023 position statement of The Menopause Society on non-hormonal management endorses, on the basis of randomized evidence, several therapeutic alternatives, namely, selective serotonin reuptake inhibitors (SSRIs), serotonin-norepinephrine reuptake inhibitors (SNRIs), gabapentin, fezolinetant, oxybutynin, cognitive behavioral therapy (CBT), and clinical hypnosis, while recommending against a range of unproven complementary interventions, including pregabalin [7].

SSRIs and SNRIs represent the most extensively studied non-hormonal pharmacological alternative to estrogen, with efficacy supported by multiple randomized trials. In the landmark trial of Loprinzi et al., conducted specifically in breast cancer survivors, venlafaxine at 75 mg daily reduced hot flash scores by approximately 60% compared with 27% for placebo [42], and subsequent placebo-controlled trials confirmed clinically meaningful benefit for paroxetine and escitalopram in menopausal populations [43,44]. The onset of action is rapid, with a measurable response within the first 1-2 weeks, in contrast to the several weeks typically required for antidepressant indications, and efficacy appears broadly comparable across subpopulations, including women with breast cancer with or without tamoxifen and women experiencing natural or surgical menopause [7,42]. Indirect comparisons suggest similar efficacy between SSRIs and SNRIs, although head-to-head trials are lacking, and citalopram and escitalopram are often preferred in practice on account of a favorable adverse effect profile and a lower risk of discontinuation symptoms [7,44].

A clinically important consideration in women receiving tamoxifen is the inhibition of CYP2D6, the enzyme that converts tamoxifen to its most potent metabolite, endoxifen; co-administration of strong CYP2D6 inhibitors, of which paroxetine is the archetype, substantially lowers plasma endoxifen concentrations [41]. A population-based cohort study further reported increased breast cancer mortality among tamoxifen users co-prescribed paroxetine, in proportion to the duration of overlap, although this association has not been demonstrated for other SSRIs [45]. Pending definitive evidence, agents with weak or negligible CYP2D6 inhibition, such as venlafaxine, citalopram, and escitalopram, are preferred in tamoxifen-treated women, whereas no comparable interaction exists between SSRIs and the aromatase inhibitors, with which co-administration is considered safe [7,41,45].

Gabapentin, an alpha-2-delta calcium channel ligand originally approved for postherpetic neuralgia and diabetic neuropathic pain, also reduces VMS, and its efficacy has been demonstrated in a large randomized placebo-controlled trial involving 420 women with breast cancer, in whom a regimen of 300 mg three times daily significantly reduced hot flash frequency and severity [46]. The drug is particularly suited to women whose symptoms predominate at night, since nocturnal episodes cluster within the first hours of sleep, and its sedative properties may act synergistically with its effect on thermoregulation while bedtime administration limits daytime exposure to adverse effects, which include somnolence, dizziness, and, rarely, respiratory or central nervous system depression, hypersensitivity reactions, and neuropsychiatric disturbances [7,28,46].

Oxybutynin, an anticholinergic agent long used for overactive bladder, has likewise demonstrated efficacy against VMS in a randomized, double-blind trial that enrolled women with and without breast cancer, the majority of whom were receiving tamoxifen or an aromatase inhibitor; both low-dose and higher-dose regimens significantly reduced hot flash frequency and severity compared with placebo, at the cost of anticholinergic effects such as dry mouth [47]. Because prolonged anticholinergic exposure has been linked in other settings to cognitive concerns in older adults, the lowest effective dose is advisable for long-term use [7,47]. Among non-pharmacological options, CBT deserves emphasis, as the randomized MENOS 1 trial demonstrated that a structured group CBT program significantly reduced the problem rating of hot flashes and night sweats in breast cancer survivors, with benefits maintained at six months, and current guidance endorses CBT and clinical hypnosis as evidence-based behavioral interventions [7,48].

Fezolinetant: A First-in-Class NK3 Receptor Antagonist

Fezolinetant, marketed as Veoza in the European Union and Australia and as Veozah in the United States, is a selective, non-hormonal NK3 receptor antagonist that competitively blocks NKB signaling on KNDy neurons in the hypothalamic arcuate nucleus, thereby restoring physiological thermoregulatory tone at the precise neural locus implicated in symptom generation [8,9]. On the strength of the BRIGHT SKY phase 3 program, comprising the SKYLIGHT 1, SKYLIGHT 2, and SKYLIGHT 4 trials, the United States Food and Drug Administration (FDA) approved fezolinetant on May 12, 2023, for the treatment of moderate-to-severe VMS associated with menopause, at a once-daily oral dose of 45 mg [9,49].

In SKYLIGHT 1, a randomized, double-blind, placebo-controlled phase 3 trial conducted across 97 sites in seven countries, fezolinetant at 30 mg and 45 mg once daily significantly reduced the frequency and severity of moderate-to-severe VMS at weeks 4 and 12 compared with placebo, with improvements detectable as early as week 1 and sustained through 52 weeks of treatment [9]. The identically designed SKYLIGHT 2 trial confirmed these findings, with least-squares mean reductions in VMS frequency exceeding 50% at week 12 in both active-treatment groups [50]. SKYLIGHT 4, a 52-week randomized safety study enrolling more than 1,800 women, demonstrated an acceptable long-term safety profile, with headache and fatigue the most frequent adverse events and no signal of endometrial pathology [51].

The safety profile of fezolinetant was subsequently refined by post-marketing experience. Following a report of serious hepatocellular-cholestatic liver injury, the FDA first added a warning in September 2024 and then, on December 16, 2024, elevated it to a boxed warning regarding the rare risk of serious drug-induced liver injury [52,53]. Current prescribing information requires measurement of hepatic transaminases and total bilirubin before initiation, monthly for the first three months of treatment, and again at six and nine months; therapy should not be started if either aminotransferase concentration or total bilirubin exceeds twice the upper limit of normal, and patients must be counseled to discontinue the drug and seek prompt medical attention should symptoms suggestive of hepatotoxicity (fatigue, nausea, pruritus, jaundice, pale stools, or dark urine) emerge [52,53]. Concomitant use with cytochrome P450 1A2 (CYP1A2) inhibitors is contraindicated, as is use in patients with known cirrhosis or severe renal impairment or end-stage renal disease [53].

Elinzanetant: Dual NK1/NK3 Receptor Antagonism

The therapeutic principle established by fezolinetant has been extended by elinzanetant, a dual neurokinin-1 (NK1) and NK3 receptor antagonist that, in addition to blocking NKB signaling on KNDy neurons, antagonizes substance P signaling through the NK1 receptor, a pathway implicated in cutaneous vasodilatation and in the regulation of sleep [10]. In the twin phase 3 trials OASIS 1 and OASIS 2, once-daily elinzanetant 120 mg significantly reduced the frequency and severity of moderate-to-severe VMS at weeks 4 and 12 compared with placebo, with onset of benefit within the first week and accompanying improvements in sleep disturbance and menopause-related quality of life [10]. The 52-week OASIS 3 trial confirmed durable efficacy and long-term tolerability, with mild headache, fatigue, and somnolence among the most common adverse events and without a serious hepatic safety signal during the trial [54]. On October 24, 2025, the FDA approved elinzanetant, marketed as Lynkuet, for moderate-to-severe VMS due to menopause, following earlier authorizations in the United Kingdom, Canada, Australia, and Switzerland; in contrast to fezolinetant, its United States labeling carries no boxed hepatic warning [55].

Neurokinin Receptor Antagonism in Women With Breast Cancer

The application of neurokinin receptor antagonism to breast cancer survivors is of particular clinical interest, because VMS are among the most common adverse reactions to adjuvant endocrine therapy, are frequently more severe than those accompanying natural menopause, particularly in younger women receiving concomitant GnRH analogues, and contribute materially to endocrine therapy discontinuation, with adverse consequences for recurrence and survival [11]. Because these agents act directly on central thermoregulatory pathways and do not require estrogen replacement to achieve symptom control, they are of particular therapeutic interest in women with hormone receptor-positive disease, for whom menopausal hormone therapy is contraindicated [7,11].

The first pivotal evidence in this population comes from OASIS 4, a randomized, double-blind, placebo-controlled phase 3 trial that enrolled 474 women aged 18-70 years who were experiencing at least 35 moderate-to-severe VMS episodes per week while receiving endocrine therapy for the treatment or prevention of hormone receptor-positive breast cancer [11]. At week 12, the mean change from baseline in the daily frequency of moderate-to-severe VMS was -7.8 episodes in the elinzanetant group and -4.2 episodes in the placebo group; the model-adjusted least-squares mean difference between groups was -3.4 episodes (95% confidence interval: -4.2 to -2.5; p < 0.001), with a significant separation already evident at week 1; key secondary endpoints demonstrated parallel improvements in sleep disturbance and menopause-related quality of life, and the 52-week safety profile (headache, fatigue, and somnolence being most frequent) was consistent with the earlier OASIS trials [11]. These results, published in the New England Journal of Medicine in 2025, represent the first demonstration that neurokinin-targeted therapy is effective for endocrine therapy-induced VMS, while the ongoing extension phase of OASIS 4 is collecting additional long-term safety data [11].

Recent subgroup analyses of OASIS 4 suggest that the efficacy of elinzanetant is maintained across women receiving tamoxifen or aromatase inhibitors, with or without concomitant ovarian function suppression, with reductions in VMS frequency and severity observed across all treatment subgroups. However, these post hoc analyses were not powered for formal comparisons between endocrine therapy subgroups and therefore do not establish differential or equivalent treatment effects between them. Evidence also remains limited regarding whether treatment response differs according to the underlying mode of ovarian failure, including natural menopause versus chemotherapy-induced ovarian failure [56].

Fezolinetant is being evaluated in the same population in two dedicated studies: HIGHLIGHT 1 (ClinicalTrials.gov identifier NCT06440967), an Astellas-sponsored phase 3 trial in women with stage 0-III hormone receptor-positive breast cancer receiving adjuvant endocrine therapy [57], and FLASH-Breast (NCT06917313), a randomized phase 2 trial [58]. The investigator-initiated phase 2 VENT trial (NCT06617455) was terminated early because of poor accrual [59]. Until these ongoing trials report, the efficacy and safety of fezolinetant specifically in breast cancer survivors remain to be established, and its use in this population is not supported by dedicated prospective efficacy data. Its non-hormonal mechanism and lack of CYP2D6-mediated interaction that complicates the use of certain antidepressants with tamoxifen provide a rationale for further evaluation in this setting, while the mandated hepatic monitoring requirements remain an important safety consideration [7,41,53].

Critical Synthesis and Future Perspectives

When the available evidence is considered as a whole, important differences between the available therapeutic approaches emerge. MHT remains the most effective treatment for VMS but is precluded in breast cancer survivors [5,6]. Antidepressants, gabapentin, and oxybutynin offer moderate benefit and remain valuable, particularly where cost or comorbidity favors them, but each carries class-specific liabilities (CYP2D6 interaction and sexual dysfunction for certain antidepressants, sedation for gabapentin, and anticholinergic burden for oxybutynin), and none addresses the underlying neuroendocrine disturbance [42,45-47]. The neurokinin receptor antagonists, by contrast, act directly on the KNDy circuit responsible for symptom generation, reduce symptom frequency within one week, and improve sleep and quality of life, and they are the only class for which pivotal, population-specific evidence in endocrine therapy-treated patients with breast cancer now exists [9-11].

Several limitations of the available evidence should nevertheless be considered. Placebo responses in VMS trials are characteristically large, frequently approaching a 40%-50% reduction in symptom frequency, which complicates the translation of trial effect sizes into everyday expectations and precludes reliable cross-trial comparison between drug classes in the absence of head-to-head studies [11,50]. The hepatotoxicity signal that prompted the fezolinetant boxed warning, although rare, imposes a monitoring burden that may limit uptake and demands vigilance in populations, such as breast cancer survivors, already exposed to hepatically metabolized therapies, while the apparent absence of a comparable hepatic signal with elinzanetant awaits confirmation in larger post-marketing experience [52,54]. The long-term oncological safety of sustained neurokinin pathway blockade in women with a history of hormone receptor-positive malignancy also remains to be established. This will require longer follow-up in breast cancer-specific populations, with systematic assessment of recurrence and new primary breast cancer events, continued evaluation of potential interactions with endocrine therapy, and surveillance extending beyond the relatively short treatment periods of current VMS trials. The durability of symptom control beyond one year, cost and equitable access, and efficacy in women with chemotherapy-induced ovarian failure also remain important evidence gaps.

## Conclusions

VMS represent an important challenge in the care of women with breast cancer, with meaningful implications for quality of life and the overall experience of long-term treatment. Their management requires therapeutic approaches that provide effective symptom relief while remaining compatible with the specific considerations of breast cancer survivorship. Neurokinin receptor antagonism represents a promising addition to the non-hormonal therapeutic landscape and offers a more targeted approach to symptom management. As clinical experience with this drug class continues to evolve, treatment should remain individualized, taking into account symptom burden, safety considerations, concomitant therapies, patient preferences, and overall oncological care. Further research will help clarify the long-term role of neurokinin receptor antagonists and their optimal position alongside established non-hormonal treatment strategies.
